# Supplementary material for: Temporal Dimensions of Job Quality and Gender: Exploring Differences in the Associations of Working Time and Health between Women and Men
Source: Int J Environ Res Public Health. 2022 Apr 7;19(8):4456. doi: 10.3390/ijerph19084456 (PMC9029241; doi:10.3390/ijerph19084456)
Supplement: Supplementary file 1 [file ijerph-19-04456-s001.zip › ijerph-1613496-supplementary.pdf]

Table S1: Descriptives of variables in Study 1 (EWCS)

	Overall	Men	Women
Negative impact of work on health	0.25	0.27	0.23
Health problems in the past 12 months	2.27	2.12	2.42
Sustainable work	0.72	0.74	0.70
Subjective well-being	3.41	3.46	3.35
JQ1 Unsocial hours	82.59	81.25	84.00
JQ2 Long hours	83.33	78.56	88.35
JQ3 Flexibility and control	63.48	64.45	62.48
JQ4 Time pressure	67.41	65.77	69.13
JQ Full index	67.71	65.76	69.76
Women	0.49	0.00	1.00
Education low	0.17	0.19	0.16
Education medium	0.51	0.52	0.50
Education high	0.31	0.29	0.34
Age under 35	0.28	0.28	0.28
Age 35-49	0.39	0.39	0.39
Age 50 and older	0.33	0.34	0.32
Agriculture	0.04	0.05	0.03
Industry	0.16	0.21	0.10
Construction	0.06	0.10	0.01
Transport	0.05	0.08	0.02
Commerce	0.15	0.14	0.15
Hospitality	0.05	0.04	0.05
Financial services	0.04	0.04	0.04
Services: Information, communication, professional	0.07	0.07	0.06
Services: admin and support	0.06	0.06	0.06
Public administration and defence	0.06	0.06	0.05
Education	0.09	0.05	0.12
Health	0.12	0.04	0.20
Other services	0.07	0.05	0.10
Managers	0.06	0.07	0.04
Professionals	0.19	0.16	0.22
Technicians and associate professionals	0.14	0.14	0.15
Clerical support workers	0.10	0.07	0.14
Service and sales workers	0.19	0.14	0.25
Skilled agricultural, forestry and fish	0.03	0.04	0.02
Craft and related trades workers	0.10	0.18	0.02
Plant and machine operators, and assemblers	0.07	0.12	0.02
Elementary occupations	0.11	0.09	0.13
Austria	0.02	0.02	0.02

Belgium	0.02	0.02	0.02
Bulgaria	0.01	0.01	0.01
Croatia	0.01	0.01	0.01
Cyprus	0.00	0.00	0.00
Czech Republic	0.02	0.02	0.02
Denmark	0.01	0.01	0.01
Estonia	0.00	0.00	0.00
Finland	0.01	0.01	0.01
France	0.12	0.12	0.12
Germany	0.18	0.18	0.19
Greece	0.02	0.02	0.01
Hungary	0.02	0.02	0.02
Ireland	0.01	0.01	0.01
Italy	0.10	0.11	0.10
Latvia	0.00	0.00	0.00
Lithuania	0.01	0.01	0.01
Luxembourg	0.00	0.00	0.00
Malta	0.00	0.00	0.00
Netherlands	0.04	0.04	0.04
Poland	0.07	0.07	0.08
Portugal	0.02	0.02	0.02
Romania	0.04	0.04	0.04
Slovakia	0.01	0.01	0.01
Slovenia	0.00	0.00	0.00
Spain	0.08	0.08	0.08
Sweden	0.02	0.02	0.02
United Kingdom	0.14	0.15	0.13

Note: Weighted averages of EWCS 2015 key variables

Table S2: Descriptives of variables in Study 2 (LFS)

	Overall	Men	Women
Health problems	0.10	0.10	0.11
Serious health problems	0.07	0.07	0.08
Exposed to physical risk	0.63	0.66	0.59
Exposed to mental risk	0.45	0.43	0.47
Decide working time	0.71	0.68	0.74
Take leave	0.47	0.45	0.49
Take free hours	0.37	0.35	0.39
Expected flexibility	0.31	0.34	0.27
Available	0.24	0.26	0.21
Time pressure	0.44	0.44	0.43
Couple	0.51	0.50	0.52
Age	43.22	42.89	43.55
Qualifications: low	0.24	0.25	0.23
Qualifications: middle	0.46	0.48	0.44
Qualifications: tertiary	0.30	0.28	0.32
Agriculture and fishing	0.04	0.05	0.03
Mining	0.00	0.01	0.00
Manufacture	0.16	0.21	0.10
Utilities	0.02	0.02	0.01
Construction	0.07	0.11	0.01
Trade and repairt	0.13	0.13	0.14
Hotels/restaurants	0.05	0.04	0.05
Transport, storage, ICT	0.09	0.12	0.05
Finance	0.03	0.03	0.03
Real estate, renting, and business	0.11	0.10	0.11
Public administration and social security	0.07	0.07	0.07
Education	0.08	0.04	0.12
Health and social work	0.11	0.05	0.19
Other services	0.05	0.04	0.07
Armed forces	0.00	0.00	0.00
Managers	0.06	0.07	0.04
Professionals	0.21	0.18	0.24
Technicians and associate professionals	0.16	0.15	0.18
Clerical lsupport workers	0.10	0.06	0.14
Service and sales workers	0.16	0.11	0.23
Skilled agricultural, forestry and fishery workers	0.03	0.04	0.02
Craft and related trades workers	0.11	0.19	0.03
Plant and machine operators, and assemblers	0.07	0.11	0.03
Elementary occupations	0.09	0.08	0.10
AT	0.02	0.02	0.02

BE	0.03	0.03	0.03
BG	0.02	0.02	0.02
CY	0.00	0.00	0.00
CZ	0.02	0.02	0.02
DE	0.16	0.16	0.15
DK	0.01	0.01	0.01
EE	0.00	0.00	0.00
ES	0.11	0.11	0.11
FI	0.01	0.01	0.01
FR	0.11	0.11	0.11
GR	0.02	0.02	0.02
HR	0.01	0.01	0.01
HU	0.02	0.02	0.02
IE	0.01	0.01	0.01
IT	0.14	0.13	0.14
LT	0.01	0.01	0.01
LU	0.00	0.00	0.00
LV	0.00	0.00	0.00
MT	0.00	0.00	0.00
NL	0.04	0.04	0.04
NO	0.01	0.01	0.01
PL	0.06	0.07	0.06
PT	0.02	0.02	0.02
RO	0.05	0.05	0.05
SE	0.02	0.02	0.02
SI	0.00	0.00	0.00
SK	0.01	0.01	0.01
UK	0.07	0.07	0.08

Note: Average of LFS 2019-2020 key variables

Table S3. Logistic regression results for negative impact of work on health (odds ratios; robust standard errors in parentheses) from Study 1

	JQ1 Unsocial hours		JQ2 Long hours		JQ3 Flexibility and control		JQ4 Time pressure		JQ Full index	
	(a)	(b)	(a)	(b)	(a)	(b)	(a)	(b)	(a)	(b)
Women	1.267	1.193	1.231	1.266*	0.984	1.260	1.301	1.346*	2.534***	2.268***
	(0.205)	(0.201)	(0.171)	(0.181)	(0.134)	(0.179)	(0.209)	(0.223)	(0.558)	(0.511)
JQ1 Unsocial hours	0.986***	0.983***								
	(0.001)	(0.001)								
Women x JQ1 (comp. men x JQ1)	0.995***	0.998								
	(0.002)	(0.002)								
JQ2 Long hours			0.989***	0.988***						
			(0.001)	(0.001)						
Women x JQ2 (comp. men x JQ2)			0.996**	0.998						
			(0.002)	(0.002)						
JQ3 Flexibility and control					0.981***	0.982***				
					(0.001)	(0.002)				
Women x JQ3 (comp. men x JQ3)					0.996**	0.995**				
					(0.002)	(0.002)				
JQ4 Time pressure							0.966***	0.965***		
							(0.002)	(0.002)		
Women x JQ4 (comp. men x JQ4)							0.994**	0.995**		
							(0.002)	(0.002)		
JQ Full index									0.962***	0.958***
									(0.002)	(0.002)
Women x JQ Full (comp. men x JQ Full)									0.985***	0.989***
									(0.003)	(0.003)

Occupation and sector included as control variables	no	yes	no	yes	no	yes	no	yes	no	yes
Constant	1.421***	1.356	1.157	0.761	1.512***	1.827***	4.636***	3.585***	6.139***	4.064***
	(0.186)	(0.294)	(0.128)	(0.154)	(0.185)	(0.405)	(0.629)	(0.810)	(0.960)	(0.959)
N	34,326	33,990	34,316	33,983	34,324	33,997	34,355	34,004	34,355	34,004
Pseudo-R^2	0.047	0.071	0.045	0.070	0.053	0.075	0.094	0.117	0.085	0.109

Note: Level of significance *** p<0.01, ** p<0.05, * p<0.1. All models include education, age group and country as control variables. EWCS 2015, EU-28 countries.

Table S4. Linear regression results for health problems in the past 12 months (robust standard errors in parentheses) from Study 1

	JQ1 Unsocial hours		JQ2 Long hours		JQ3 Flexibility and control		JQ4 Time pressure		JQ Full index	
	(a)	(b)	(a)	(b)	(a)	(b)	(a)	(b)	(a)	(b)
Women	0.878***	0.838***	0.547***	0.541***	0.313***	0.377***	0.650***	0.676***	1.204***	1.205***
	(0.155)	(0.157)	(0.134)	(0.136)	(0.118)	(0.121)	(0.136)	(0.138)	(0.195)	(0.196)
JQ1 Unsocial hours	-0.008***	-0.010***								
	(0.001)	(0.001)								
Women x JQ1 (comp. men x JQ1)	-0.006***	-0.005***								
	(0.002)	(0.002)								
JQ2 Long hours			-0.010***	-0.010***						
			(0.001)	(0.001)						
Women x JQ2 (comp. men x JQ2)			-0.002	-0.001						
			(0.001)	(0.001)						
JQ3 Flexibility and control					-0.010***	-0.011***				
					(0.001)	(0.001)				
Women x JQ3 (comp. men x JQ3)					-0.000	-0.000				
					(0.002)	(0.002)				
JQ4 Time pressure							-0.027***	-0.028***		
							(0.001)	(0.001)		
Women x JQ4 (comp. men x JQ4)							-0.003*	-0.004**		
							(0.002)	(0.002)		
JQ Full index									-0.030***	-0.033***
									(0.002)	(0.002)
Women x JQ Full (comp. men x JQ Full)									-0.011***	-0.010***
									(0.003)	(0.003)
Occupation and sector included as control variables	no	yes	no	yes	no	yes	no	yes	no	yes

Constant	2.741***	2.964***	2.904***	2.855***	2.747***	3.182***	3.948***	4.017***	4.143***	4.071***
	(0.118)	(0.191)	(0.101)	(0.176)	(0.103)	(0.187)	(0.114)	(0.189)	(0.138)	(0.200)
N	35,424	35,069	35,414	35,061	35,409	35,068	35,475	35,091	35,475	35,091
R^2	0.096	0.104	0.099	0.107	0.093	0.102	0.144	0.153	0.133	0.142

Note: Level of significance *** p<0.01, ** p<0.05, * p<0.1. All models include education, age group and country as control variables. EWCS 2015, EU-28 countries.

Table S5. Logistic regression results for sustainable work (odds ratios; robust standard errors in parentheses) from Study 1

	JQ1 Unsocial hours		JQ2 Long hours		JQ3 Flexibility and control		JQ4 Time pressure		JQ Full index	
	(a)	(b)	(a)	(b)	(a)	(b)	(a)	(b)	(a)	(b)
Women	0.390***	0.407***	0.544***	0.561***	0.887	0.835	0.621***	0.570***	0.301***	0.302***
	(0.066)	(0.072)	(0.077)	(0.083)	(0.125)	(0.123)	(0.100)	(0.094)	(0.066)	(0.067)
JQ1 Unsocial hours	1.006***	1.006***								
	(0.001)	(0.002)								
Women x JQ1 (comp. men x JQ1)	1.009***	1.008***								
	(0.002)	(0.002)								
JQ2 Long hours			1.001	1.001						
			(0.001)	(0.001)						
Women x JQ2 (comp. men x JQ2)			1.004***	1.004**						
			(0.002)	(0.002)						
JQ3 Flexibility and control					1.019***	1.018***				
					(0.002)	(0.002)				
Women x JQ3 (comp. men x JQ3)					0.999	0.999				
					(0.002)	(0.002)				
JQ4 Time pressure							1.015***	1.017***		
							(0.002)	(0.002)		
Women x JQ4 (comp. men x JQ4)							1.003	1.005**		
							(0.002)	(0.002)		
JQ Full index									1.014***	1.015***
									(0.002)	(0.002)
Women x JQ Full (comp. men x JQ Full)									1.014***	1.014***
									(0.003)	(0.003)

Occupation and sector included as control variables	no	yes	no	yes	no	yes	no	yes	no	yes
Constant	0.508***	1.094	0.792**	1.540**	0.264***	0.377***	0.310***	0.569**	0.330***	0.735
	(0.071)	(0.237)	(0.093)	(0.310)	(0.033)	(0.084)	(0.043)	(0.126)	(0.055)	(0.170)
N	32,004	31,697	31,996	31,691	31,998	31,697	32,020	31,706	32,020	31,706
Pseudo-R^2	0.066	0.085	0.058	0.080	0.076	0.095	0.071	0.096	0.069	0.091

Note: Level of significance *** p<0.01, ** p<0.05, * p<0.1. All models include education, age group and country as control variables. EWCS 2015, EU-28 countries.

Table S6. Linear regression results for subjective well-being (robust standard errors in parentheses) from Study 1

	JQ1 Unsocial hours		JQ2 Long hours		JQ3 Flexibility and control		JQ4 Time pressure		JQ Full index	
	(a)	(b)	(a)	(b)	(a)	(b)	(a)	(b)	(a)	(b)
Women	-0.162**	-0.196**	-0.091	-0.118*	-0.053	-0.096	-0.250***	-0.257***	-0.283***	-0.329***
	(0.079)	(0.080)	(0.063)	(0.064)	(0.062)	(0.064)	(0.070)	(0.070)	(0.097)	(0.098)
JQ1 Unsocial hours	0.004***	0.005***								
	(0.001)	(0.001)								
Women x JQ1 (comp. men x JQ1)	0.000	0.000								
	(0.001)	(0.001)								
JQ2 Long hours			0.003***	0.004***						
			(0.000)	(0.000)						
Women x JQ2 (comp. men x JQ2)			-0.001	-0.001						
			(0.001)	(0.001)						
JQ3 Flexibility and control					0.007***	0.007***				
					(0.001)	(0.001)				
Women x JQ3 (comp. men x JQ3)					-0.001	-0.001				
					(0.001)	(0.001)				
JQ4 Time pressure							0.012***	0.012***		
							(0.001)	(0.001)		

Women x JQ4 (comp. men x JQ4)							0.001	0.001		
							(0.001)	(0.001)		
JQ Full index									0.014***	0.015***
									(0.001)	(0.001)
Women x JQ Full (comp. men x JQ Full)									0.002	0.002
									(0.001)	(0.001)
Occupation and sector included as control variables	no	yes	no	yes	no	yes	no	yes	no	yes
Constant	3.231***	3.204***	3.256***	3.310***	3.101***	2.944***	2.708***	2.751***	2.610***	2.697***
	(0.061)	(0.089)	(0.052)	(0.080)	(0.055)	(0.090)	(0.058)	(0.084)	(0.070)	(0.090)
N	35,378	35,030	35,367	35,022	35,370	35,034	35,416	35,045	35,416	35,045
R^2	0.044	0.051	0.044	0.051	0.053	0.060	0.087	0.094	0.072	0.082

Note: Level of significance *** p<0.01, ** p<0.05, * p<0.1. All models include education, age group and country as control variables. EWCS 2015, EU-28 countries.

Table S7: Logistic regression of experiencing health problems (odds ratios and standard error) from study 2

Job quality variable:	Part-time	Under-employed PT	Long hours	Shift work	Unsocial hours
Women	1.390***	1.356***	1.387***	1.330***	1.328***
	(0.0295)	(0.0274)	(0.0293)	(0.0323)	(0.0366)
Cohabiting	0.928***	0.924***	0.917***	0.919***	0.923***
	(0.0173)	(0.0172)	(0.0172)	(0.0189)	(0.0190)
Education: low	0.950*	0.950*	0.952*	0.941*	0.934**
	(0.0263)	(0.0263)	(0.0269)	(0.0298)	(0.0297)
Education: high	0.927***	0.929***	0.926***	0.965	0.950*
	(0.0221)	(0.0222)	(0.0223)	(0.0257)	(0.0254)
Age: <34	0.564***	0.565***	0.571***	0.561***	0.563***
	(0.0144)	(0.0144)	(0.0147)	(0.0154)	(0.0155)
Age: >55	1.258***	1.256***	1.252***	1.255***	1.258***

	(0.0235)	(0.0233)	(0.0235)	(0.0265)	(0.0266)
Job Quality indicator	0.995	0.940	1.372***	1.230***	1.247***
	(0.0476)	(0.0882)	(0.0475)	(0.0454)	(0.0364)
Women X job quality	0.906*	1.004	0.970	1.193***	1.128***
	(0.0492)	(0.106)	(0.0596)	(0.0575)	(0.0439)
Constant	0.122***	0.120***	0.111***	0.108***	0.0940***
	(0.0181)	(0.0178)	(0.0166)	(0.0170)	(0.0149)
Observations	441,928	441,928	429,960	370,271	370,271

Note: estimated from logistic regression controlling for country, occupation and sector fixed effects, weighted. Odds ratios and standard errors are shown. LFS 2020
**** p<0.01, ** p<0.05, * p<0.1*

Table S8: Logistic regression of experiencing serious health problems (odds ratios and standard error) from study 2

Job quality variable:	Part-time	Under-employed PT	Long hours	Shift work	Unsocial hours
Women	1.476***	1.427***	1.456***	1.385***	1.398***
	(0.0367)	(0.0334)	(0.0358)	(0.0395)	(0.0447)
Cohabiting	0.907***	0.903***	0.894***	0.906***	0.908***
	(0.0194)	(0.0192)	(0.0193)	(0.0215)	(0.0215)
Education: low	0.965	0.967	0.970	0.956	0.948
	(0.0285)	(0.0285)	(0.0291)	(0.0325)	(0.0323)
Education: high	0.895***	0.897***	0.895***	0.940**	0.923**
	(0.0252)	(0.0253)	(0.0254)	(0.0295)	(0.0292)
Age: <34	0.497***	0.498***	0.505***	0.500***	0.502***
	(0.0155)	(0.0156)	(0.0159)	(0.0168)	(0.0169)

Age: >55	1.373***	1.375***	1.371***	1.369***	1.370***
	(0.0288)	(0.0287)	(0.0290)	(0.0326)	(0.0327)
Job Quality indicator	1.132**	1.000	1.367***	1.208***	1.199***
	(0.0584)	(0.107)	(0.0536)	(0.0524)	(0.0409)
Women X job quality	0.816***	0.978	1.012	1.258***	1.123***
	(0.0481)	(0.115)	(0.0710)	(0.0700)	(0.0506)
Constant	0.0781***	0.0777***	0.0707***	0.0669***	0.0598***
	(0.0133)	(0.0132)	(0.0122)	(0.0121)	(0.0110)
Observations	441,928	441,928	429,960	370,271	370,271

Note: estimated from logistic regression controlling for country, occupation and sector fixed effects, weighted. Odds ratios and standard errors are shown. LFS 2020
**** p<0.01, ** p<0.05, * p<0.1*

Table S9: Logistic regression of being exposed to physical risk (odds ratios and standard error) from study 2

Job quality variable:	Part-time	Under-employed PT	Long hours	Shift work	Unsocial hours
Women	0.919***	0.898***	0.919***	0.910***	0.906***
	(0.0117)	(0.0108)	(0.0115)	(0.0126)	(0.0144)
Cohabiting	0.955***	0.950***	0.949***	0.957***	0.962***
	(0.0112)	(0.0111)	(0.0113)	(0.0124)	(0.0124)
Education: low	0.878***	0.877***	0.877***	0.896***	0.884***
	(0.0171)	(0.0171)	(0.0175)	(0.0201)	(0.0198)
Education: high	0.801***	0.806***	0.804***	0.828***	0.809***
	(0.0116)	(0.0117)	(0.0118)	(0.0133)	(0.0130)
Age: <34	0.844***	0.839***	0.849***	0.825***	0.831***

	(0.0120)	(0.0119)	(0.0122)	(0.0127)	(0.0128)
Age: >55	0.946***	0.933***	0.934***	0.963***	0.964**
	(0.0119)	(0.0117)	(0.0119)	(0.0137)	(0.0137)
Job Quality indicator	0.638***	0.699***	1.364***	1.651***	1.482***
	(0.0183)	(0.0337)	(0.0323)	(0.0432)	(0.0269)
Women X job quality	1.178***	1.185***	0.961	1.161***	1.125***
	(0.0391)	(0.0659)	(0.0389)	(0.0416)	(0.0282)
Constant	6.289***	5.891***	5.537***	5.698***	4.611***
	(0.526)	(0.492)	(0.470)	(0.508)	(0.416)
Observations	435,554	435,554	423,710	364,633	364,633

Note: estimated from logistic regression controlling for country, occupation and sector fixed effects, weighted. Odds ratios and standard errors are shown. LFS 2020
**** p<0.01, ** p<0.05, * p<0.1*

Table S10: Logistic regression of being exposed to mental risk (odds ratios and standard error) from study 2

Job quality variable:	Part-time	Under-employed PT	Long hours	Shift work	Unsocial hours
Women	1.049***	1.019*	1.057***	1.015	1.042***
	(0.0122)	(0.0111)	(0.0119)	(0.0128)	(0.0151)
Cohabiting	0.955***	0.951***	0.945***	0.954***	0.960***
	(0.00988)	(0.00981)	(0.00991)	(0.0109)	(0.0110)
Education: low	0.805***	0.802***	0.807***	0.836***	0.828***
	(0.0120)	(0.0120)	(0.0124)	(0.0143)	(0.0143)
Education: high	1.117***	1.122***	1.117***	1.155***	1.133***
	(0.0146)	(0.0146)	(0.0148)	(0.0166)	(0.0164)

Age: <34	0.800***	0.796***	0.811***	0.789***	0.791***
	(0.0101)	(0.0100)	(0.0104)	(0.0108)	(0.0109)
Age: >55	0.832***	0.824***	0.821***	0.842***	0.848***
	(0.00932)	(0.00920)	(0.00933)	(0.0106)	(0.0108)
Job Quality indicator	0.722***	0.943	1.798***	1.590***	1.671***
	(0.0176)	(0.0394)	(0.0364)	(0.0321)	(0.0253)
Women X job quality	1.049*	0.911*	0.939*	1.182***	1.084***
	(0.0301)	(0.0445)	(0.0351)	(0.0345)	(0.0235)
Constant	0.970	0.926	0.822***	0.918	0.672***
	(0.0703)	(0.0672)	(0.0607)	(0.0703)	(0.0525)
Observations	433,285	433,285	421,572	362,559	362,559

Note: estimated from logistic regression controlling for country, occupation and sector fixed effects, weighted. Odds ratios and standard errors are shown. LFS 2020
**** p<0.01, ** p<0.05, * p<0.1*

Table S11: Logistic regression of gender differences in job quality as odds ratios (s.e.) from study 2

	Part-time		Under-employed PT		Long hours		Shift work		Unsocial hours	
	(a)	(b)	(a)	(b)	(a)	(b)	(a)	(b)	(a)	(b)
Women	5.294***	3.637***	3.028***	1.915***	0.343***	0.400***	0.962***	0.776***	0.820***	0.649***
	(0.0733)	(0.0579)	(0.0693)	(0.0527)	(0.00547)	(0.00736)	(0.0123)	(0.0125)	(0.00814)	(0.00824)
Cohabiting	1.107***	1.172***	0.718***	0.753***	1.191***	1.149***	0.898***	0.925***	0.878***	0.905***
	(0.0149)	(0.0164)	(0.0164)	(0.0179)	(0.0197)	(0.0198)	(0.0126)	(0.0142)	(0.00946)	(0.0106)
Education: low	1.389***	1.109***	1.601***	1.175***	0.994	0.923***	0.839***	0.794***	0.999	0.862***
	(0.0235)	(0.0204)	(0.0406)	(0.0324)	(0.0194)	(0.0195)	(0.0154)	(0.0162)	(0.0144)	(0.0140)

Education:										
high	0.651***	0.860***	0.535***	0.860***	1.195***	1.176***	0.401***	0.724***	0.696***	0.970**
	(0.00956)	(0.0157)	(0.0145)	(0.0281)	(0.0202)	(0.0254)	(0.00614)	(0.0147)	(0.00789)	(0.0147)
Age: <34	1.271***	1.191***	1.434***	1.357***	0.671***	0.673***	1.194***	1.102***	1.165***	1.040***
	(0.0199)	(0.0195)	(0.0365)	(0.0363)	(0.0138)	(0.0144)	(0.0187)	(0.0190)	(0.0144)	(0.0141)
Age: >55	1.479***	1.461***	0.970	0.936**	1.018	0.992	0.778***	0.778***	0.809***	0.817***
	(0.0230)	(0.0238)	(0.0276)	(0.0277)	(0.0182)	(0.0186)	(0.0139)	(0.0151)	(0.0107)	(0.0117)
Constant	0.135***	0.0910***	0.0132***	0.00232***	0.184***	0.915	0.323***	0.0609***	1.129***	2.242***
	(0.00342)	(0.0224)	(0.000865)	(0.00190)	(0.00563)	(0.137)	(0.00872)	(0.0291)	(0.0241)	(0.453)
N	473,172	473,172	465,785	465,785	458,922	458,922	394,823	394,823	394,825	394,825

Note: Estimated through logistic regression, weighted. (a) includes country fixed effects, (b) includes country, sector, and occupation fixed effects and the combination of sector and occupation. LFS 2020

*** p<0.01, ** p<0.05, * p<0.1

Table S12: OLS regression of gender differences in job quality (s.e.) from study 2

	Decide working time		Take leave		Take free hours		Expected flexibility		Available		Time pressure	
	(a)	(b)	(a)	(b)	(a)	(b)	(a)	(b)	(a)	(b)	(a)	(b)
Women	0.0750***	0.0385***	0.0433***	0.0137***	0.0545***	0.0215***	-0.0776***	-0.0557***	-0.0648***	-0.0603***	-0.0248***	-0.00950***
	(0.00166)	(0.00179)	(0.00145)	(0.00161)	(0.00152)	(0.00165)	(0.00178)	(0.00202)	(0.00145)	(0.00164)	(0.00137)	(0.00155)
Cohabiting	-0.0324***	-0.0253***	-0.00911***	-0.00694***	-0.0160***	-0.0119***	0.00656***	0.00215	0.0181***	0.0136***	0.0127***	0.00924***
	(0.00183)	(0.00170)	(0.00157)	(0.00151)	(0.00165)	(0.00157)	(0.00195)	(0.00191)	(0.00160)	(0.00156)	(0.00149)	(0.00147)

Education: low	0.0165***	0.0117***	0.00521**	0.000963	0.0143***	0.0101***	-0.0433***	-0.0337***	-0.0562***	-0.0368***	-0.0528***	-0.0358***
	(0.00230)	(0.00220)	(0.00207)	(0.00210)	(0.00220)	(0.00219)	(0.00240)	(0.00244)	(0.00186)	(0.00188)	(0.00201)	(0.00206)
Education: high	-0.125***	-0.0550***	-0.0279***	-0.0122***	-0.0545***	-0.0235***	0.0886***	0.0486***	0.0831***	0.0298***	0.0524***	0.0242***
	(0.00191)	(0.00225)	(0.00165)	(0.00196)	(0.00172)	(0.00200)	(0.00207)	(0.00254)	(0.00173)	(0.00209)	(0.00154)	(0.00188)
Age: <34	0.0715***	0.0610***	0.0258***	0.0203***	0.0482***	0.0413***	-0.0198***	-0.0169***	-0.0389***	-0.0335***	-0.0267***	-0.0255***
	(0.00207)	(0.00196)	(0.00184)	(0.00180)	(0.00195)	(0.00187)	(0.00228)	(0.00225)	(0.00185)	(0.00182)	(0.00174)	(0.00173)
Age: >55	-0.0479***	-0.0412***	-0.0338***	-0.0341***	-0.0337***	-0.0321***	-0.0204***	-0.0206***	-0.0160***	-0.0171***	-0.0390***	-0.0355***
	(0.00223)	(0.00205)	(0.00185)	(0.00179)	(0.00194)	(0.00184)	(0.00225)	(0.00221)	(0.00186)	(0.00182)	(0.00175)	(0.00173)
Constant	0.700***	0.328***	0.455***	0.394***	0.380***	0.190***	0.362***	0.575***	0.253***	0.478***	0.461***	0.515***
	(0.00355)	(0.0256)	(0.00334)	(0.0218)	(0.00351)	(0.0173)	(0.00412)	(0.0287)	(0.00324)	(0.0270)	(0.00297)	(0.0213)
N	465,866	465,866	460,224	460,224	462,151	462,151	460,540	460,540	460,703	460,703	461,539	461,539
R2	0.075	0.225	0.035	0.099	0.053	0.154	0.056	0.095	0.064	0.114	0.073	0.102

Note: Estimated through OLS, weighted. (a) includes country fixed effects, (b) includes country, sector, and occupation fixed effects and the combination of sector and occupation. LFS 2020

*** p<0.01, ** p<0.05, * p<0.1
